# Correlation Between Severity of Symptoms and Quality of Life in Patellofemoral Pain Syndromes: A Cross-Sectional Study

**DOI:** 10.7759/cureus.49094

**Published:** 2023-11-20

**Authors:** Tushara Nair, G. Palani Kumar

**Affiliations:** 1 College of Physiotherapy, Sumandeep Vidyapeeth (Deemed to be University), Vadodara, IND

**Keywords:** quality of life, function, severity of symptoms, pain, patellofemoral pain syndromes

## Abstract

Background

Patellofemoral pain syndrome (PFPS) is one of the conditions frequently encountered by musculoskeletal physiotherapists. The intensity and severity of pain and symptoms seen in PFPS affect the quality of life (QOL). This concept has not yet been investigated with the use of reliable and valid scales. Hence, the objective of this study is to find out the correlation between the severity of symptoms and QOL in patients with PFPS.

Methodology

In this cross-sectional study, 63 patients with PFPS, aged between 40 and 60 years, who visited Sir Sayajirao General Hospital, Vadodara, from December 2018 to June 2019, were included. The questionnaires included in the study comprised the Numerical Pain Rating Scale (NPRS), the Knee Osteoarthritis Outcome Survey-Patellofemoral Subscale (KOOS-PF), and the Short Form-36 (SF-36). Spearman’s rank correlation coefficient was used to find the correlation.

Results

NPRS was found to have a fairly positive correlation with the functional scale KOOS-PF, with a *P*-value of 0.008. Among the eight components of SF-36, Physical Function and General Health scores exhibited a fairly positive correlation with NPRS, with p-values of 0.016 and <0.001, respectively. However, no correlation was observed between NPRS and the other components of SF-36.

Conclusions

This study concluded that patients with PFPS with different levels of pain exhibit different functional and QOL limitations. Furthermore, studies investigating the causes of the negative correlation should be conducted to have a better understanding of QOL in PFPS.

## Introduction

Patellofemoral pain syndromes (PFPS) are most frequently encountered by physiotherapists dealing with musculoskeletal problems and represent one of the most common causes of anterior knee pain among individuals aged 15 to 60 years [1]. It is an umbrella term that states that structural damage exceeds the articular cartilage [2]. The major cause of PFPS is abnormal biomechanics that is muscular weakness and tightness, leading to malpositioning of the patella and abnormal patellar tracking [2-6].

The World Health Organization (WHO) defines quality of life (QOL) as an individual’s understanding of life with respect to one’s beliefs [7]. Patients with PFPS are prone to depression and kinesiophobia, which affects psychosocial functioning and QOL [8,9,10].

To the best of our knowledge, no study has been conducted yet in India comparing the severity of symptoms and QOL in patients with PFPS using appropriate, reliable, and valid scales. Therefore, this study aims to bridge this gap in the available evidence in the literature.

## Materials and methods

This study is part of an interventional study *Effect of Yoga Therapy to Improve Function and Level of Pain in Patients with Patellofemoral Pain Syndromes - A Randomized Controlled Trial*. The ethical approval for this study was obtained from the Institutional Ethics Committee for Human Research (IECHR), Medical College and Sir Sayajirao General (SSG) Hospital, Baroda.

Study design

A cross-sectional, time-bound study was conducted for a duration of six months from December 2018 to June 2019 in the Outdoor Physiotherapy Department of SSG Hospital, Vadodara. The sample size was calculated to be 63 using the following formula: *n*= 4*pq*/*L*^2^, where the prevalence was found to be 15% with an allowable error of 9%. The patients diagnosed with PFPS by an orthopedic surgeon or a physiotherapist fulfilling the inclusion and exclusion criteria were included in the study.

Inclusion criteria

Patients in the age group of 40-60 years experiencing non-traumatic anterior or retro-patellar knee pain during at least two of the following activities: prolonged sitting, stairs, squatting, running, kneeling, and hopping/jumping for at least one month, and reporting pain on patellar palpation as well as during stepping onto a 25-cm step or during a double-leg squat, were included in the study.

Exclusion criteria

Individuals with a recent history (within three months) of knee surgery, a history of patellar dislocation/subluxation, or clinical evidence of meniscal lesion, ligamentous instability, traction apophysitis around the patellofemoral complex, patellar tendon pathology, and any severe neurological, cardiopulmonary, or musculoskeletal impairments other than patellofemoral pain syndromes were excluded from the study. Patients on corticosteroid medication use and pregnancy were also excluded.

Procedure

All the subjects diagnosed with PFPS by an orthopedic surgeon or physiotherapist based on the inclusion and exclusion criteria and willingness to participate were included in the study [4]. Written and informed consent was taken from all the participants. After that a thorough clinical history was taken and a complete physical and functional physiotherapy examination was done in each case. The Numerical Pain Rating Scale (NPRS), the Knee Osteoarthritis Outcome Score-Patellofemoral Subscale (KOOS-PF), and the Short Form-36 (SF-36) were the questionnaires that were used.

Variables and instruments:

The pain was assessed using self-reported NPRS which has good to excellent reliability with an Intra-class Correlation Coefficient (ICC) value of 0.88 (95% CI, 0.81-0.92), and the concurrent validity compared to that of Visual Analogue Scale (VAS) was 0.910. The scores are grouped into three 0-3, 4-6, and 7-10 which indicate mild, moderate, and severe levels of pain respectively [11].

The patellofemoral pain and osteoarthritis symptoms were assessed using the KOOS-PF subscale in the form of an interview. It includes 11 items that focus on pain during activities that load the patellofemoral joint. The higher the score the lesser the disability and the lower the score the greater the disability. It has good reliability with an ICC value of 0.86 and good concurrent validity compared to that of the Anterior Knee Pain Scale (AKPS) with r = 0.74 [12].

The Medical Outcomes Study Questionnaire Short Form-36 Health Survey (SF-36) was used to assess the QOL. It includes eight dimensions that are physical functioning, social functioning, role limitations (physical problems), role limitations (emotional problems), pain, mental health, vitality, and general health problems. Lower scores indicate greater disability and higher scores indicate lesser disabilities. It was found to have good reliability and validity with Cronbach’s coefficient> 0.85 [13].

The statistical analysis was done using IBM SPSS Statistics 20.0 software. The baseline data was analyzed using the Kolmogorov-Smirnov test and correlation was found using the Spearman’s rank correlation coefficient. The coefficient value of greater than 0.75 indicates good to excellent correlation, 0.5 to 0.75 indicates moderate correlation and 0.25 to 0.5 indicates fair correlation [14].

## Results

**Figure 1:**
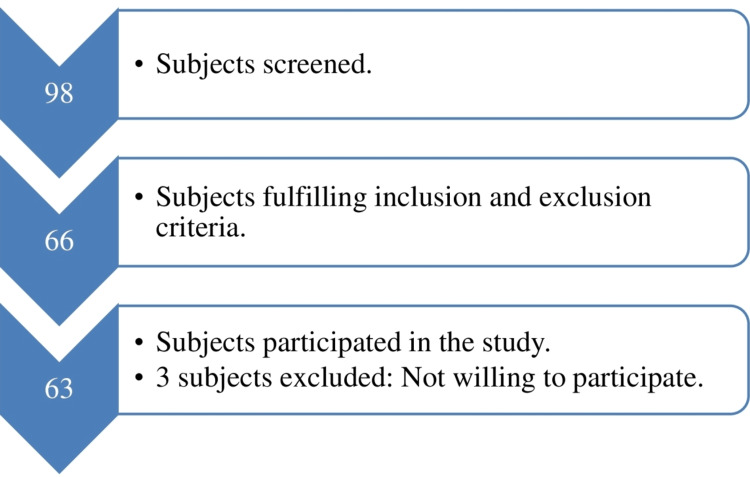
Flow diagram indicating data collection. Image credit: Tushara Nair.

A total of 98 patients diagnosed with PFPS by an orthopedic surgeon or a physiotherapist referred to the Outdoor Physiotherapy Department of SSG Hospital, Vadodara, were screened. A total of 63 patients were included based on inclusion and exclusion criteria and their willingness to participate. Three patients were not willing to participate because of time constraints. The flowchart depicting the samples included in the study is described in Figure 1.

The normality of the baseline data, including age and BMI, was assessed using the Kolmogorov-Smirnov Test (Table 1). There was no significant difference in the baseline data among the subjects, and the data were found to follow the normal distribution curve.

**Table 1:** Baseline characteristics. SD, standard deviation; P, probability; BMI, body mass index

Variable	Mean ± SD	*P*-value	Kolmogrov-Smirnov statistic (*D*)
Age (years)	51.35 ± 6.77	0.048	0.1691
BMI (kg/m^2^)	26.17 ± 2.53	0.704	0.08628

The demographic data regarding gender distribution and the joints affected by PFPS are mentioned in Table 2.

**Table 2:** Demographic data. *N*, number

Demographic data	Variable	N
Gender distribution	Female	37
	Male	26
Joints affected	Unilateral	43
	Bilateral	20

Table 3 indicates the correlation of NPRS with the functional scale KOOS-PF and SF-36. The correlation of NPRS with KOOS-PF and components of SF-36 was determined using the Spearman rank correlation coefficient. 

**Table 3:** Correlation between pain, function, and quality of life. NPRS, Numerical Pain Rating Scale; KOOS-PF, Knee Osteoarthritis Outcome Survey-Patellofemoral Subscale; SF-36, Short Form-36

Variable	Correlation with NPRS	
	*R* (rho)	*P*-value
KOOS-PF	-0.33	0.008
SF-36 Physical Function	-0.301	0.016
SF-36 Role Limitation Due to Physical Problem	-0.132	0.301
SF-36 Role limitation Due to Emotional Problem	-0.146	0.25
SF-36 Energy	-0.133	0.298
SF-36 Emotional Well-Being	-0.008	0.952
SF-36 Social Function	-0.064	0.617
SF-36 Pain	-0.148	0.246
SF-36 General Health	-0.467	<0.001

NPRS was found to have a fair correlation with the functional scale KOOS-PF with a *P*-value of 0.008. Among the eight components of SF-36, Physical Function and General Health scores were found to have a fair correlation with NPRS with a *P*-value of less than 0.05, whereas no correlation was found between other components of SF-36 and NPRS.

**Figure 2:**
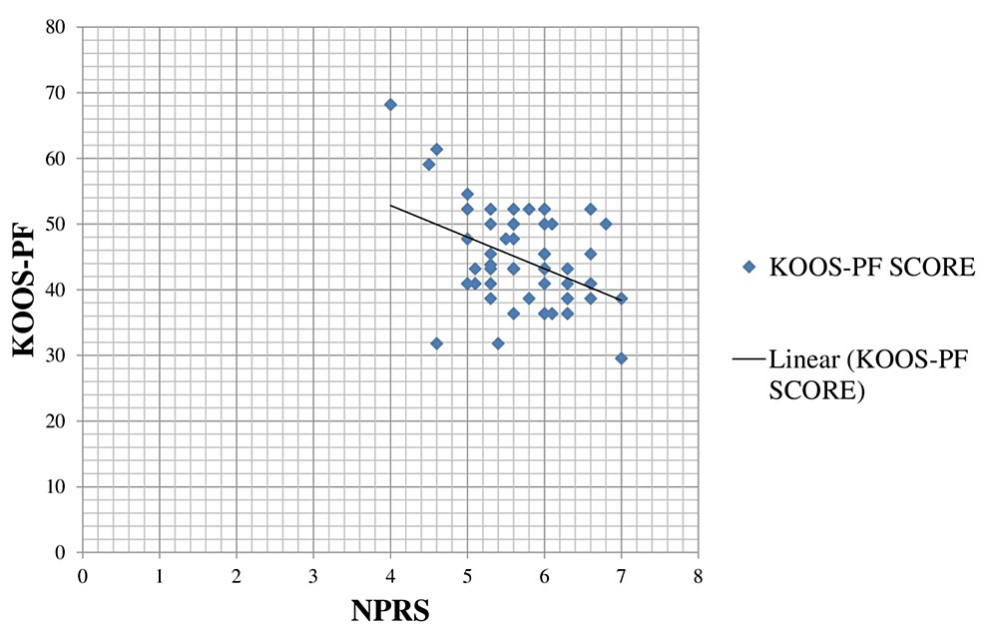
Scatter plot diagram showing a correlation between NPRS and KOOS-PF. NPRS, Numerical Pain Rating Scale; KOOS-PF, Knee Osteoarthritis Outcome Survey-Patellofemoral subscale

The scatter diagram indicating the correlation between NPRS and KOOS-PF is shown in Figure 2, which describes that as the pain level (NPRS) increases, the functional level and disability score (KOOS-PF) decreases.

**Figure 3:**
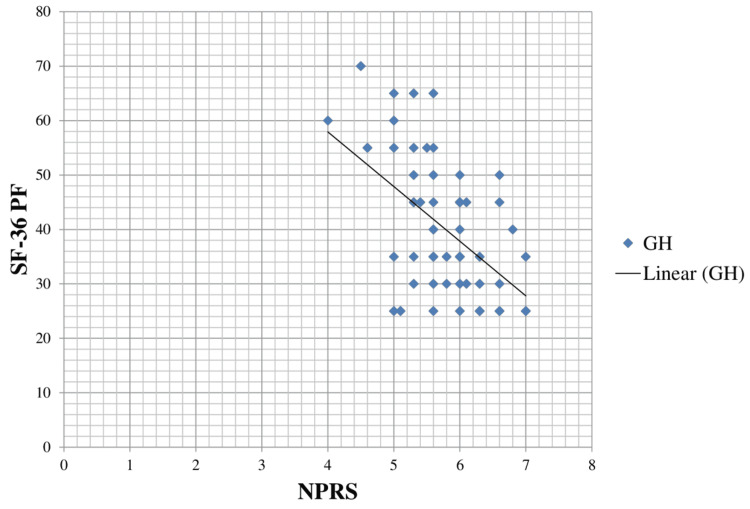
Scatter plot diagram showing a correlation between NPRS and SF-36 PF. NPRS, Numerical Pain Rating Scale; SF-36 PF, Short Form-36 Physical Function

The scatter diagram indicating the correlation between NPRS and SF-36 Physical Function (SF-36 PF) is shown in Figure 3, which describes that as the pain level (NPRS) increases, the SF-36 PF score decreases.

**Figure 4:**
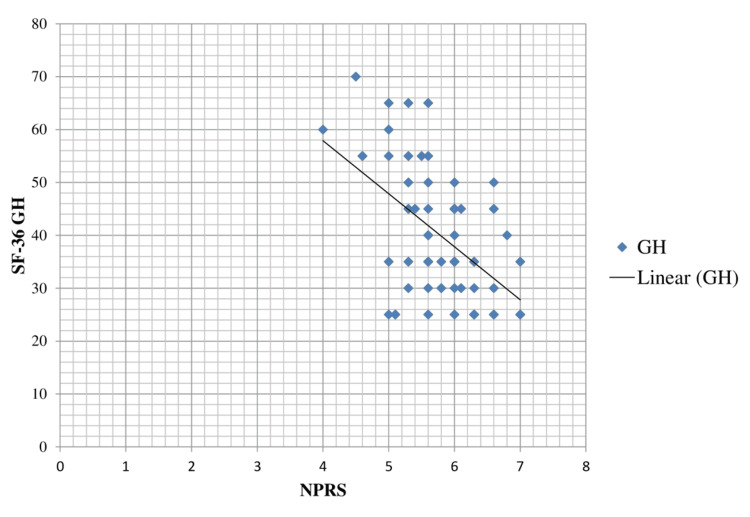
Scatter plot diagram showing a correlation between NPRS and SF-36 GH. NPRS, Numerical Pain Rating Scale; SF-36 GH, Short Form-36 General Health

The scatter diagram indicating the correlation between NPRS and SF-36 General Health (SF-36 GH) is shown in Figure 4, which describes that as the pain level (NPRS) increases, the SF-36 GH score decreases.

## Discussion

In this study, the relationship between the level of pain on the functional level and QOL was studied in PFPS patients. The level of pain was assessed using NPRS. In 2018, Young et al. studied the psychometric properties of NPRS and Neck Disability Index (NDI) on 107 patients and found that NPRS had moderate reliability [11]. Therefore, NPRS was used for recording the pain level of patients. 

The Kujala Anterior Knee Pain Scale (AKPS) is the most commonly used scale for patellofemoral pain, but the components of osteoarthritis were not included [12,13]. To overcome this problem, in 2018, Crossley et al. developed a KOOS-PF subscale for patellofemoral pain and osteoarthritis, which was found to have good reliability and validity [13]. According to the inclusion criteria of this study, patellofemoral osteoarthritis patients were also included; therefore, the KOOS-PF subscale developed by Crossley et al. in 2018, focusing on the arthritic symptoms as well, was used.

In 2013, Sinha et al. concluded that the Medical Outcome Survey (MOS) SF-36 had good validity and reliability for use in India [14]. Therefore, the aforementioned scale was used in this study to assess QOL.

The results concluded that there was a negative correlation between NPRS and KOOS-PF, and the strength of the association was fair. As NPRS increases, the functional level and disability of the patient decrease; therefore, the score of KOOS-PF decreases. Therefore, the coefficient was found to be negative. Similarly negative correlation was found between NPRS and SF-36 Physical Function and General Health components with fair strength of association. Aw et al. conducted a study on 233 patients with knee osteoarthritis and concluded that patients with pain sensitization had poorer QOL, supporting our study [15].

No correlation was found between NPRS and other components of SF-36: Role Limitation Due to Physical Problems and Emotional Problems, Energy, Emotional Well-Being, Social Function, and Pain.

Shah et al. studied QOL in the elderly population of Ahmedabad, India, in 2017. Of the total subjects, 42.8% had joint pain as a comorbidity, whereas the overall QOL was found to be good to excellent [16]. As described by WHO, QOL is the individual’s understanding of life with respect to their plan, beliefs, and standards [17]. Indians are spiritually rooted and tend to live a happy and content life despite the difficulties. Therefore, the QOL of the Indian population remains to be good to excellent. Similarly in this study as well the pain level was not associated with some components of SF-36: Individual Role Limitations, Energy, Emotional Well-Being, and Social Function. The majority of the subjects had unilateral joint involvement, which could also be one of the reasons for the negative results.

This study concludes that QOL is not always related to the severity of pain as many other factors influence QOL. The results derived from this study are difficult to generalize to the whole population as the sample size was small, the method of sampling was convenient, and data were collected from a single hospital. Therefore, further more detailed studies should be conducted with a larger sample size at multiple sites and with a stronger methodology to find out the factors affecting QOL. 

## Conclusions

PFPS is one of the disorders most commonly encountered by physiotherapists. Patients with PFPS with a different level of pain exhibit different functional and QOL limitations. This study concluded that there is a fairly positive correlation between pain level (NPRS) and functional level (KOOS-PF), as well as between NPRS and PF and GH components, whereas no correlation was found between NPRS and other components on SF-36, as the Indian population tend to continue their daily activities despite the pain.
